# Intracardiac Thrombosis during Adult Liver Transplantation

**DOI:** 10.1155/2013/618352

**Published:** 2013-07-24

**Authors:** Marina Moguilevitch, Carlene Broderick

**Affiliations:** Department of Anesthesiology, Montefiore Medical Center, Albert Einstein College of Medicine, Bronx, NY 10463, USA

## Abstract

Intracardiac thrombosis (ICT) and pulmonary embolism (PE) during adult liver transplantation are rare but potentially lethal complications. They are often overlooked because of significant diagnostic challenges. The combination of hemodynamic compromise and transesophageal echocardiography (TEE) findings allows for correct diagnosis. A large variety of putative risk factors for ICT and PE have been suggested, but these events are considered to be multifactorial. There are different proposed treatment modalities for these devastating complications. Unfortunately, in spite of growing knowledge in this area, intraoperative and postoperative mortalities remain very high. The retrospective nature of the study of these events makes the case reports extremely valuable.

## 1. Introduction

Orthotopic liver transplantation (OTL) is a major surgical procedure which usually involves significant blood loss secondary to coagulation disturbances typical of end stage liver disease. In addition, hyperfibrinolysis can occur intraoperatively, leading to more bleeding complications.

In contrast to bleeding coagulopathies, intraoperative thrombotic complications are less frequently encountered but deserve special attention because of possible life threatening events.

We are reporting a case of intracardiac thrombosis during OLT presented as a sudden cardiac arrest during the neohepatic stage.

Patient's family permission was obtained to publish this case report.

## 2. Case Presentation

A 67-year-old, 78 kg, 176 cm man with a history of hypertension, diabetes mellitus, and decompensated liver disease secondary to hepatitis C virus (HCV) with portal hypertension, ascites, and hepatic encephalopathy presented for retransplantation with an MELD score of 31. He had undergone liver transplantation 18 months previously that was complicated by a severe recurrence of HCV infection, partially responding to therapy with interferon and ribavirin. 

Relevant laboratory studies revealed 82 g/L hemoglobin, 129.6 *μ*mol/L creatinine, 165.87 *μ*mol/L total bilirubin, 2.7 INR, 26.7 sec. prothrombin time (PT), 56 sec. partial thromboplastin time (PTT), 59,000 × 10^9^/L platelets. Preoperative dobutamine stress test demonstrated normal left and right ventricular functions without evidence of ischemia.

**Figure 1:**
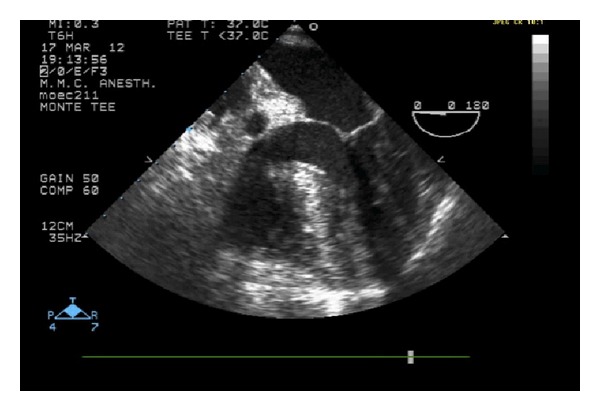
Transesophageal echocardiogram showing thrombus in the left ventricle.

After preoxygenation, anesthesia was induced with 2 mg midazolam, 250 mcg fentanyl, 60 mg propofol, and 10 mg vecuronium. After intubation, two additional 16 g peripheral intravenous catheters, a radial arterial catheter and an 8.5 French introducer with pulmonary artery catheter, were placed. The dissection phase was complicated by significant blood loss, leading to transfusion of multiple units of blood, fresh frozen plasma, and cryoprecipitate. Venovenous bypass (VVB) with a heparin-coated circuit was initiated because the patient was not able to tolerate caval cross-clamping. There was no significant or prolonged hemodynamic instability after graft reperfusion. Ninety minutes after discontinuation of VVB, a sudden precipitous drop of the blood pressure with concomitant elevations of central venous pressure and pulmonary artery pressure was noticed. This circulatory collapse led to pulseless electrical cardiac activity. CPR protocol was initiated (including infusions of epinephrine, vasopressin, and norepinephrine). A transesophageal echo probe was placed, and the study revealed a dilated right ventricle and a collapse of the left ventricle with a thrombus fluctuating under the aortic valve (Figure 1). No thrombus was seen in the pulmonary vessels.

The resuscitation efforts were unsuccessful, and the decision was made to administer tissue plasminogen activator (t-PA) 50 mg intravenously. Shortly after t-PA administration, the thrombus in the left ventricle dissolved and right ventricle function normalized. Unfortunately, dissolution of the thrombus did not lead to the restoration of normal hemodynamics despite the administration of high doses of pressors. After eighty minutes, resuscitation efforts were stopped.

## 3. Discussion

Since the first report of a thromboembolic event during OLT has been published in 1988 [1], pulmonary thromboembolism (PTE) and ICT have been major causes of intraoperative morbidity and mortality during adult OLT. The incidence of reported PTE and ICT ranges from 1.2% [2] to 6.2% [3]. A review of the literature [4, 5] failed to identify the etiology of this condition; treatment recommendations are inconsistent.

The hemostatic system is a balance between coagulation, anticoagulation, and fibrinolysis. Liver transplantation poses potential massive blood loss due to the patient's preexisting hypocoagulable state, collateral circulation caused by portal hypertension, and major surgery involving multiple vascular anastomoses. Recently, it has been shown that the balance between pro- and antithrombotic factors synthesized by the liver may be reset to a lower level in patients with chronic liver disease [6]. Liver failure causes a reduction of both pro- and anticoagulative factors production. Therefore, under normal conditions, the patients with liver disease do not bleed spontaneously. Bleeding can be caused by additional factors like infection, vascular abnormality, portal hypertension, or mechanical distraction during invasive procedures. 

Any disturbance in the delicate balance between coagulation, anticoagulation, and fibrinolysis can lead to fatal bleeding or thrombotic complication. It is known that the defect in platelet number and function is balanced by the elevated plasma concentration of von Willebrand factor (vWf) and factor VIII, which are not synthesized by the liver. Moreover, we can see a decrease in the production of all procoagulant factors synthesized by the liver as well as of anticoagulant proteins: protein C, protein S, protein Z, antithrombin, and heparin factor II. In addition, the synthesis of all proteins involved in fibrinolysis is decreased in patients with liver disease. The levels of tissue plasminogen activator (tPA) and plasminogen activator inhibitor (PAI-1) are elevated, secondary to reduced clearance by the liver. PAI-1 and tPA have extrahepatic origin. Moreover, coagulation tests like PT measures only procoagulant factors, and INR was developed and validated only for those patients on anticoagulation therapy. 

The complexity of hemostatic problems in patients with end stage liver disease (ESLD) undergoing liver transplantation and the inability of routine tests to reflect defects in the pro- and anticoagulant system make the anesthetic care of these patients more challenging.

There have been multiple attempts to identify risk factors and diagnostic predictors for potentially fatal thrombotic complications during OLT. The most recent retrospective review [5] analyzed 495 consecutive, isolated OLTs from deceased donors. The standard technique was VVB without antifibrinolytic prophylaxis. Among the preoperative recipient risk factors were an increased incidence of portal hypertension with ascites, low serum albumin levels, and preoperative hospitalization especially with ventilator support. In terms of intraoperative factors, the group with thrombotic complications had higher baseline pulmonary artery pressures, more hemodynamic instability, increased level of serum lactate, and a flat-line thromboelastogram (TEG). The diagnostic criteria for PTE and ICT were the combination of hemodynamic compromise and TEE findings of intravascular or intracardiac thrombosis. Heparin, tissue plasminogen (tPA), and hemodynamic support with vasoactive drugs were the medications of choice to treat this potentially lethal complication. Intraoperative and in-hospital mortalities after thrombotic complications reached 30% and 45%, respectively. 

 ICT remains a highly lethal and multifactorial complication, which is still very difficult to prevent and treat.
